# ‘By identifying myself as Métis, I didn’t feel safe…’: Experiences of navigating racism and discrimination among Métis women, Two-Spirit and gender diverse community members in Victoria, Canada

**DOI:** 10.1177/13558196231188632

**Published:** 2023-07-12

**Authors:** Willow Paul, Renée Monchalin, Monique Auger, Carly Jones

**Affiliations:** 1 Research Assistant, School of Social Work, 8205 University of Victoria, Victoria, BC, Canada; 2 Assistant Professor, School of Public Health and Social Policy, 8205 University of Victoria, Victoria, BC, Canada; 3 Dalla Lana School of Public Health, University of Toronto, Toronto, ON, Canada; 4 PhD Candidate, School of Public Health and Social Policy, 8205 University of Victoria, Victoria, BC, Canada; 5 Research Assistant, School of Social Work, 8205 University of Victoria, Victoria, BC, Canada

**Keywords:** Métis, racism, health services

## Abstract

**Objective:**

Racism acts as a major barrier to accessing health services for Indigenous communities in Canada, often leading to delayed, avoided or lack of treatment altogether. The Métis population is uniquely positioned in urban settings, as they experience discrimination from both Indigenous and mainstream health and social services due to Canada’s long colonial history that is ongoing. Yet, Métis are often left out of discussions regarding racism and health service access. This study explores the experiences of racism and health service access among Métis peoples in Victoria, British Columbia.

**Methods:**

We allied a conversational interview method to explore and understand experiences of self-identifying Métis women, Two-Spirit and gender diverse people (*n* = 24) who access health and social services in Victoria. Data analysis followed Flicker and Nixon’s six-stage DEPICT model.

**Results:**

In this paper, we share the experiences of racism and discrimination of those who accessed health and social services in Victoria, British Columbia Such experiences include passing as White, experiencing racism following Métis identity disclosure and witnessing racism. Passing as White was viewed as a protective factor against discrimination as well as harming participants’ sense of identity. Experiences of racism took the form of discriminatory comments, harassment and mistreatment, which influenced the willingness of disclosing Métis identity. Witnessing racism occurred in participants, personal and professional lives, negatively impacting them in indirect ways. Each experience of racism had a negative influence on participants’ wellbeing and shaped their experience of accessing health and social services.

**Conclusions:**

Métis people confront racism and discrimination when attempting to access health and social services through first-hand experiences, witnessing and/or avoidance. While this study contributes to the all too often unacknowledged voices of Métis in Canada, there is a continued need for Métis-specific research to accurately inform policy and practice.

## Introduction

Racism against Indigenous peoples is pervasive in Canada. From interpersonal interactions to structural racism enacted through systems and institutions, racism is a common experience faced among First Nations, Métis and Inuit peoples, collectively referred to as the Indigenous population. As Loppie and de Leeuw explain, racism is to be understood as a lived experience, something that occurs to individuals, communities and nations through interactions with people and structures in everyday life.^1^ Racism has been described as a significant determinant of Indigenous peoples’ health and serves as a major barrier when accessing health services, leading to delayed, lack of and/or avoidance of treatment.^2^

In British Columbia (B.C.), Canada, the 2020 report *In Plain Sight* explored Indigenous peoples’ experiences with racism when accessing health services.^3^ It found that Indigenous peoples do not feel safe accessing the provincial health care system. Indigenous respondents were less likely to have positive health care outcomes and over 80% reported experiencing racism or discrimination when accessing health services.^3^ The report demonstrates the severity of Indigenous-racism within B.C. health services; however, the distinct experiences of Métis people accessing services continue to be largely unknown. The lack of Métis voices is problematic as Métis people account for over a third of the Indigenous population in both Canada and in B.C., and they experience severe disparities in health determinants and outcomes compared to the non-Indigenous Canadian population due to a long and ongoing history of colonialism and assimilation.^4–6^ This has had direct implications on health and social service access, resulting in Métis people being ‘caught betwixt and between’ First Nations and mainstream services.^7^

Métis people's health service access experiences are often left out of discourses surrounding racism and service access and this study seeks to address this gap.^8^ It is situated in the city of Victoria, B.C., which is home to over 6900 self-identifying Métis people, accounting for 38% of the Indigenous population in the city.^4^ Available evidence has highlighted the lack of culturally safe services for the Métis community in B.C., but little is known about Victoria itself.^9–11^ This study explores the experiences of racism and health service access among Métis women, Two-Spirit and gender diverse people in the southernmost part of Vancouver Island on the traditional and unceded territory of the Lekwungen and WSÁNEĆ nations in Victoria, in an effort to improve access to culturally safe health services.

## Methods

This study expands on multiple years of exploring Métis people’s experiences with health services in urban settings.^8^ Indigenous led and community-driven at all stages, we used a qualitative approach involving interviews with people self-identifying as Métis woman and/or Two-Spirit and/or trans-identifying woman. Prospective participants had to be 19 years or older, having lived, worked and/or received health and/or social services in Victoria within 12 months of the interview. Recruitment was achieved through a poster designed by a local Métis artist and shared to a Facebook group (Métis Nation of Greater Victoria). Twenty-five eligible participants agreed to be interviewed, but one potential participant was unable to coordinate a time for their interview so did not participate in the study, leaving 24 participants. Prior to contacting participants, we sought consent and guidance by a local and prominent Métis Elder living in Victoria who informed the project goals, recruitment plan, interview guide and the thematical analysis before sharing the final results.

Data collection coincided with the COVID-19 pandemic, and interviews were therefore conducted by video or audio calls using Zoom during December 2020 and January 2021. Interviews were approximately 60 minutes in length and conducted in private, using headphones to ensure confidentiality. Métis research assistants (CJ, WP) led interviews using a conversational interview method.^12^ This method promoted a natural way for participants’ stories to develop while also avoiding the rigidity of a more formal interview guide. It encouraged relationship building and is aligned with Métis ways of sharing knowledge through storytelling and informal ‘kitchen-table’ conversations.^13^

The conversational interview guide was based on a study engaging with Métis women in Toronto,^8^ and refined by the project’s Métis Elder to better capture Victoria’s unique Métis community context. It consisted of ten open-ended questions that focused on Métis identity; experiences with health and social services; racism and discrimination; COVID-19; and recommendations for improving access to health and social services. This paper reports on findings from the racism and discrimination theme, which explored two questions: ‘…Can you think of a time that you have been treated poorly or unfairly because you are Métis’? and ‘How did this impact your overall health and wellbeing’? Each participant was provided a $95 honorarium for their time which they were e-transferred at the beginning of each interview. Participants were also mailed a thank you card designed by the local Victoria Métis artist as well as a COVID-19 appropriate face mask. The face masks were designed by two different Indigenous artists who identified as Métis and Anishinaabe.

Interviews were transcribed, with transcripts anonymised and each participant assigned with a local plant medicine in the Michif language that would be used as a pseudonym for their quotes.^14^ All but one of the participants consented to having their direct quotes used for analysis alongside their pseudonym.

Data analysis followed Flicker and Nixon’s six-stage DEPICT model.^15^ This required each research team member to read each transcript to identify major themes to support codebook development. All four authors were responsible for coding transcripts using the codebook within NVivo software, using holistic and descriptive coding.^16,17^ To improve rigour, all four authors were part of the thematic analysis, with two authors responsible for coding each transcript. Authors were well versed in Indigenous methodologies and qualitative methods and self-identified as young Métis women with shared familial and lived experiences. Eight participants wanted to review their transcripts and we shared each transcript along with their individualised themes. This process further enhanced rigour and validity, determining if the research team’s identified themes accurately reflected participant experiences. Participants made corrections or deletions to their transcripts which were mailed back to the research team and integrated as advised. Team members generated a summary and supporting quotes for each theme, which were then reviewed by the team who collaboratively agreed on edits. These were shared with the project’s Métis Elder to ensure that we embodied and represented participants’ feedback in an appropriate way.

### Ethics review

The project received ethics approval from the University of Victoria (application no. 20-0469).

## Findings

Interview transcripts identified three overarching themes regarding experiences of racism and discrimination: passing as White; experiencing racism and discrimination; and witnessing racism, although it is important to note that experiences of racism and discrimination are inextricably tied to one another. We report on each theme in turn.

### Passing as White

Sixteen Métis women, Two-Spirit and gender diverse community members shared experiences of being ‘White passing’ or ‘White presenting’ to describe the experience of appearing White and having others perceive them as such. For example, “I don’t look very Indigenous. I’m a White-passing person. And so (…) earlier I didn’t even feel like I could say that I was Indigenous because I didn’t feel like I had enough in me and now that I’m realising what it is, that’s not it, I just don’t look like it, so if I don’t identify people have no idea” *(Mazhaan; Nettle)*. Participants shared that passing as White comes with a unique set of privileges and challenges.

It’s interesting that a lot of Métis people, they can be very White passing I guess and … I’ve always thought that was kind of a privilege and I think that maybe that’s a product of growing up in a White supremacist society… loss and the feeling that you don’t belong anywhere. That comes along with it too. So, it’s not a privilege in that sense in that it is accompanied by a lot of pain and, you know, a lot of Métis kids were in residential schools as well, and then they were … isolated from both ends of their heritage. So that was an interesting perspective that I learned. *(lii tii’d mashkek; Labrador Tea)*

Whenever I’m asked if I’m First Nation, Métis, or Inuit, I never disclose because I’m afraid... I am White passing and I have the privilege in being able to not disclose my identity and I’ve heard way too often the experiences of folks who don’t have that privilege, how they’ve been treated, so I tend not to say I’m Métis. And if I’m looking for Métis-specific services, again, that question of identity comes into play, so is someone going to be asking you for a card at the door and what does that look like and how am I going to be treated on the other end? (*li pchi boom; Wild mint*)

The experience of being White passing is not without its challenges. Métis participants reported negative impacts such as a lost sense of identity, tensions of ‘prizing’ Whiteness, rooted in White Supremacy, feelings of inadequacy, and fear when disclosing Métis identity. The relationship between Métis identity and being White passing is a complex one for participants to navigate: “But everyone assumes I’m White and then that frustrates me even more because I’m not. I’m Métis” *(La gratelle, Balsam)*. While challenging, participants also considered their White appearance to be a privilege as it usually meant that they were not discriminated against and would actively avoid disclosing their Métis identity to avoid discrimination. Fourteen participants noted that they had never experienced racism, and that their White privilege could be a protective factor against racism in health care and elsewhere:

In terms of health, I’ve never experienced any kind of judgement or any racism or anything... I don’t have my status. Everything is self-identified. And so, it never comes up on any of my medical forms. People don’t usually assume... because I’m White-passing and experience a lot of White privilege and benefit from that, I have never had any kind of experiences in the health system related to that, which I’m thankful for. *(lii grenn bleu; Blueberry)*

Within the larger health care system, I’m pretty anonymous, you know, nobody ever asks me whether I’m Indigenous or non-Indigenous … I don’t generally experience overt racism because … I’m visibly pretty White so for the most part, most people don’t make that assumption. When I do disclose, I often get the stuff about… we start getting into the blood quantum garbage and that’s when I start going, “I don’t want to discuss this with you. I don’t want to get into that whole business.” And so I usually just shut down and say, “I don’t want to talk about this with you.” So, for the most part, then I just walk into the (appointment saying) “Can we just deal with this as, you know, White people to White people?” *(lii groo zel; Goose berry)*

For Métis people in Victoria, passing as White comes along with a unique set of challenges and privileges that they navigate to avoid discrimination. Challenges include being subject to having their identity assumed or questioned. This experience has negative impacts on a sense of identity for Métis participants as identity may often be associated with one’s appearance. Intertwined with these challenges unique to being White passing is the privileged experience of being able to navigate and avoid discriminatory treatment while accessing services.

### Experiences of racism and discrimination

Ten participants spoke about having experiences of racism and discrimination after they self-identified as Métis. This was inclusive of experiencing rude jokes and comments surrounding inaccurate stereotypes about Métis people and other Indigenous groups, sexual harassment, and mistreatment in terms of the care that they received. Métis participants experienced racism and discrimination while in the community and while accessing various services beyond direct health services, such as transport.

One of my most horrendous experiences was with a cab driver and I had just come from a Missing and Murdered Indigenous Women’s circle. And there was some community organising taking place. And I had gotten into the cab and I was a bit shooken up, emotionally... I was heading home. And I was living in Victoria; I disclosed I was Métis at that time, and the guy drove me through where women were working on the street and started talking about how they got what they deserved... the whole way home. And I asked to get off early... and I walked the rest of the way. I was so scared, and I told my partner at that time, I was so afraid of that person driving that cab. And I called the cab company, and they never did follow up with me with a report. So, you know, there’s reasons we don’t access those spaces too. It’s not just the health care system, it’s... you know, systemic racism that exists across a lot of services. And, living in a rural area has... has compounded that because buses don’t even run sometimes to these areas. *(li pchi boom; Wild mint)*

Experiences included workplace racism within the health services.

When I was at work was the most difficult time in terms of the racism. So ironically, I was leading a major project in culturally response service delivery for Aboriginal people. And I was completely ostracised. So I was the only Aboriginal person on my team. And I, I understand that it was difficult for the people on my team to go through this intense sort of training and transformative journey. And I became the target of that… their discomfort. So that was… that was just awful, like it was just absolutely awful because I was also their leader. So I had to lead the project and walk in a really good way. So here I am, supposed to be, you know, trying to model how my ancestors would navigate this and in the face of outright violence, right? Like just racist, terrible, awful… like literally would not acknowledge my presence. So I’m in a … cubicle of four people and my boss comes out and nobody would speak to me. Like nobody. They all spoke amongst each other, nobody would acknowledge my presence. *(lii groo zel; Goose berry)*

Participants spoke about the overwhelming sense of fear that they hold in terms of being identified as Métis because of experiences of racism and discrimination linked to disclosure of Métis identity. The fear of disclosing identity comes from not only individual experiences with racism and discrimination, but also the frequency of racism experienced by Indigenous peoples:

By identifying myself as Métis, I didn’t feel safe. I felt like I could encounter some kind of systemic discrimination possibly by identifying myself as Métis… for Indigenous people, it’s such a common thing that you just don’t know for sure. *(enn fleur di pwayzoon; Hemlock)*

And then I stopped telling my friends and everything. Like, my good, close friends, yes. But people who didn’t really know me (would say things like), “Hey, she’s getting extra help because she’s Métis. How come I don’t get the help when I’m a White person?” Or... that’s the other one. “You’re not Métis. You don’t look Métis. How can you be Métis? That makes no sense.” Or, “You’re only two-thirds. How can you be Métis? That makes no sense. Why would the government help you?” *(Enn fleur daan loo; Waterlilly)*

As a protective strategy against racism, seven participants indicated that they strategically chose to disclose their Métis identity based on their discernment of whether the service delivery was safe enough to do so.

For the most part, when I go to health services I don’t always identify as Métis. It really depends on who I’m meeting with and, I mean, that individual is in a health service capacity... but whether or not I feel a connection or rapport, that’s what it depends on. But I would identify more as Métis if I felt comfortable that being Indigenous is not going to change how I’m treated, and that I’ll feel safe. *(lii frayz; Strawberry)*

I make that decision selectively. For some people, I’m very proud and I’ll tell them quite openly. And, for other people, I’m very careful about how I reveal that because I experience that racism all the time. *(lii groo zel; Goose berry)*

Participants disclosing Métis identity left them subject to racist and discriminatory treatment, such as discriminatory comments and mistreatment while accessing services. This has led to participants intentionally disclosing their Métis identity to avoid racist treatment.

### Witnessing racism

Five participants spoke about witnessing racism and discrimination experienced by friends and family members due to being ‘visibly’ Indigenous. The experience of witnessing racism is interwoven with being able to pass as White. Witnessing racism among family and community members resulted in participants feeling angry, stressed and excluded.

I’m very White presenting so I haven’t experienced any racism on account of the fact that I’m Métis. Other family members, totally different. You know, when I hear instances of my grandpa, his experience with racism, totally different... and … obviously that doesn’t land well. That doesn’t feel great for me either but really makes me quite angry. And he experiences it to this day. *(li pisaanlii; Dandelion)*

My experiences were very ‘normal’. And I’m very aware that I am White passing and I don’t look like I’m Métis, or Indigenous and so I’m very aware that I am probably treated as a white person, which everyone should be treated the same way. I don’t think I have been discriminated against, based on my appearance, at least in... as it pertains to appearing Indigenous. But it’s interesting, ‘cause my dad has many experiences. He looks... he appears very Métis and he has many experiences accessing health services where he’s been treated very poorly based on that, when he’s been seeking pain medication or anything like that. And I’ve heard from other friends, and I’m just... I’m very aware that I don’t have that experience in medical services. *(bouquets rouge; Fireweed)*

One participant who works in the health care system spoke to witnessing racism while on shift, and how, based on someone’s physical features, they were treated differently.

…So, say, a 20-year-old girl who’s blonde comes in intoxicated, staff might joke about, “Oh, she had a fun night”. But if somebody the same who’s Indigenous comes in, it’s more like, “Ugh, typical”, you know? (...) Some people access services more often than others and there are familiar faces that you do see regularly and... I have no medical training so my perception is limited. But I mean, I’ve heard staff say, “Oh, you know, it’s okay. They’re drunk. They can sleep it off”. And whether or not that’s the case, (…) I just think, ‘Oh, you never know, you know, you hear these stories and…’ *(enn rooz faroosh; Wild Rose)*

Participants often spoke about the direct and indirect harms they had experienced through witnessing racism, describing these as emotionally draining: “there’s nothing I can do, so that feeling of powerlessness, you know, in the moment, and the environment, you kind of have to, out of survival, brush it off, tough skin, but then you come home and it sometimes feels heavy” *(enn rooz faroosh; Wild Rose)*. Because of these experiences, participants noted that their motivation for participating in this research was linked to their family members’ experiences as racialised Métis people.

I know people in my family who have had pretty traumatic experiences, like Métis people in my family.... you just see there’s not as much care that goes into it and so it brought a lot of stress onto my mom who then had to figure everything out herself and then communicate with doctors for what like this, yeah, it was just very frustrating to see. *(li grachaw; Burdock)*

Whether witnessing racism in their professional life or their family life, participants reported the experience of witnessing racism to have negative impacts on their identity and wellbeing.

## Discussion

The stories shared by Métis participants illustrate the nuances of identity, positionality and appearance, which intersect with experiences of racism and discrimination when accessing health and social services in Victoria, B.C. Participants’ experiences of racism and discrimination were shaped by intersecting systems of power, privilege, and oppression and the structural systems of power that negatively impacted experiences varied. Overall, sexism and racism were the predominant oppressive systems that impacted participants when accessing health and social services. In acknowledging Métis peoples intersectional experiences and realities, health and social services in Victoria could be more responsive and better tailored to meet the needs of Métis community members.^18^

Participants spoke to passing as White as a privileged position since it allowed them to avoid poor treatment or discrimination by not being physically identified as Métis. Socially, the term ‘White passing’ refers to a non-White person’s ability to look like a White person, generally because they have lighter skin tones.^19^ Yet this is problematic because it centres Whiteness as the default race and something to strive for. Richardson and Seaborn^20^ explain that, for Métis people, “passing refers to the act of appearing to belong to another culture, without being noticed as different” (p. 123). Often, ‘passing’ is done strategically in order to promote personal or family safety and overall wellness.^21^ Historically, many Métis families used this strategy within non-Indigenous or First Nations communities, using ‘the politics of silence and denial’^22^ or, alternatively, to preserve Métis culture in families, but only doing so in secret.^23^ While necessary tactics for avoiding racist treatment, these strategies have contributed misunderstandings around identity and belonging.^22,23^ Discrimination, lateral violence and othering, rooted within colonial views of mixed-blood inferiority, present ongoing challenges for Métis people, who are continually marginalised within the dominant Western culture.^8^ This strategic disclosure of identity was evident among participants who were able to ‘pass’ in order to avoid discriminatory treatment.

Indigenous peoples with non-stereotypical features are often assumed that they are less ‘Indian’ and challenged on how much ‘Indian blood’ they have.^8^ This notion is based on colonial blood quantum beliefs, an idea that can be manipulated depending on the race in question. For example, comparing the experiences of Black and Indigenous peoples, Wolfe^24^ describes a ‘one drop rule’ as “(A)ny amount of African ancestry, no matter how remote, and regardless of phenotypical appearance, makes a person Black. For Indians, in stark contrast, non-Indian ancestry compromised their indigeneity, producing ‘half-breeds’, a regime that persists in the form of blood quantum regulations. As opposed to enslaved people, whose reproduction augmented their owners’ wealth, Indigenous people obstructed settlers’ access to land, so their increase was counterproductive” (p. 388).

This has been problematic for Métis people. It is troublesome for a group of people whose identity is centred around a type of ‘mixedness’ as opposed to being viewed as ‘bi-cultural’ as is the case for others in many other colonised countries.^20^

Building from their discussion around the position of White passing, participants spoke to experiencing racism and discrimination and how that dictated when or if they would self-identify as Métis when accessing health and social services. The fear of racist treatment weighs heavily as a factor when deciding to disclose Métis identity, an observation also reported by Métis in Toronto.^8^ When the threat of discrimination looms over people’s heads while accessing services, the stakes for disclosing Métis identity are high. The issue of disclosure in the face of discrimination is not exclusive to those who receive services. We found that Métis health service providers working in mainstream settings encountered racism and discrimination from fellow non-Indigenous service providers while at work, an observation also documented by Monchalin et al.’s research in Toronto.^8^ These experiences create an unsafe environment for both Indigenous people accessing mainstream services, as well as the Métis women working within these services.

Sites of racism extend beyond the direct receipt of health and social services however, and include, for example, public transport, which is frequently used by the Métis community to access services, and often presents the only means for doing so. Similarly, accessing services requires engaging with a wider group of people, such as receptionists who manage appointments and payments. Participants identified these broader service spaces as places where they experienced racism and discrimination. This suggests that interventions targeting racism should adopt a broader definition of health and social services when considering the spaces where racism occurs.

We identified witnessing racism as a further core theme in our research, and the subject of passing also emerged here. With passing being a common experience among participants, whether unintentional or as a survival strategy, this option has allowed for safety in an otherwise racist society.^20^ However, it is limited in that “passing does not protect the individual from the internal wounds received through witnessing racism towards other Métis people, particularly other family members and loved ones”^20^ (p.124), an impact also reported by participants in this study, with feelings of upset and anger that family, friends or other community members who present as more visibly Indigenous were treated poorly.

Monchalin et al.^8^ also reported witnessing racism and discrimination among Métis participants working in mainstream health or social services in Toronto. This was seen to be the result of other service providers misjudging the Métis participants as non-Indigenous, leading to presumptions that it was appropriate to make racist comments about Indigenous patients in the presence of these Métis participants.^8^ The complexities of being a White passing Indigenous person is enmeshed in the experience of witnessing other Indigenous peoples be discriminated against, negatively affecting the wellbeing of those who endure witnessing such events. Whether it be past experiences or the anticipation of racist treatment, racism is a major barrier to health care access, leading to delayed or no treatment, both with damaging effects.^2^ In this way, witnessing racism can contribute to the anticipation of racist treatment, with negative impacts on accessing care.

### Strengths and limitations

We used a random purposeful sampling approach to recruit Métis participants;^25^ yet, the experiences of racism and discrimination documented in this study may not be representative of the entire Métis community in Victoria, although we succeeded in recruiting a comparatively large sample. Conducting interviews using Zoom created flexibility for conducting conversations, but it only allowed those who had access to a phone or computer to participate. Despite this possible limitation, our research contributes to the limited literature on Métis people’s experiences with and navigation of racism and discrimination, in particular Victoria, B.C. Similar observations were reported for Métis peoples in Toronto and they may be applicable in other urban Canadian contexts.^8^

### Implications for policy and practice

Our research has several important implications for the design of policies seeking to improve access to culturally safe health services for the Métis peoples.^11^ First, our study participants highlighted the need for *Métis-specific cultural safety training* to educate service providers about Métis peoples’ unique experiences, rather than the pan-Indigenous approach to cultural safety training that is currently offered. Cultural safety is derived from scholarship of Maori nurses in response to observed disparities in the quality of health care and health outcomes between Maori and non-Maori individuals.^26^ Cultural safety moves beyond cultural awareness, cultural sensitivity and cultural competency by challenging “power imbalances, institutional discrimination, colonisation and colonial relationships as they apply to health care”.^27, p. 3^ Cultural safety training has the potential to target the culture of health care as a site for change and to accomplish this while grounded in Métis knowledge and practices.^28,29^ Any such training should be evaluated to ensure service providers are accountable.^28^ Second, study participants emphasised the importance of having *non-judgemental service providers.* This includes service providers who are trauma-informed, willing to listen, who will believe Métis people and not minimise their experiences. A trauma-informed approach is critical given what we know about how trauma can negatively impact individual health and how individuals respond to health interventions, which can result in delaying service access.^30^

## Conclusion

Despite accounting for a third of the Indigenous population in Victoria, Métis people continue to confront racism and discrimination when attempting to access health and social services through first-hand experiences, witnessing and/or avoidance. These experiences are rooted within the long and ongoing history of colonialism and assimilation targeted at Métis populations in Canada.^5,6,8^ Our research underscores the importance of understanding and addressing racism and discrimination as a determinant of Métis peoples’ health. The experiences faced by Métis women, Two-Spirit and gender diverse community members in Victoria provide a glimpse into Métis people’s experiences across Canada, and demonstrate the need for Métis voices to be continually prioritised.
